# Emerging Sonodynamic Therapy‐Based Nanomedicines for Cancer Immunotherapy

**DOI:** 10.1002/advs.202204365

**Published:** 2022-11-27

**Authors:** Yunrong Yang, Jia Huang, Min Liu, Yige Qiu, Qiaohui Chen, Tianjiao Zhao, Zuoxiu Xiao, Yuqi Yang, Yitian Jiang, Qiong Huang, Kelong Ai

**Affiliations:** ^1^ Department of Pharmacy Xiangya Hospital Central South University Changsha Hunan 410008 P. R. China; ^2^ National Clinical Research Center for Geriatric Disorders Xiangya Hospital Central South University Changsha Hunan 410008 P. R. China; ^3^ Xiangya School of Pharmaceutical Sciences Central South University Changsha Hunan 410078 P. R. China; ^4^ Hunan Provincial Key Laboratory of Cardiovascular Research Xiangya School of Pharmaceutical Sciences Central South University Changsha Hunan 410078 P. R. China

**Keywords:** cancer immunotherapy, immune adjuvants, immune checkpoint blockade therapy, immunogenic cell death, sonodynamic therapy

## Abstract

Cancer immunotherapy effect can be greatly enhanced by other methods to induce immunogenic cell death (ICD), which has profoundly affected immunotherapy as a highly efficient paradigm. However, these treatments have significant limitations, either by causing damage of the immune system or limited to superficial tumors. Sonodynamic therapy (SDT) can induce ICD to promote immunotherapy without affecting the immune system because of its excellent spatiotemporal selectivity and low side effects. Nevertheless, SDT is still limited by low reactive oxygen species yield and the complex tumor microenvironment. Recently, some emerging SDT‐based nanomedicines have made numerous attractive and encouraging achievements in the field of cancer immunotherapy due to high immunotherapeutic efficiency. However, this cross‐cutting field of research is still far from being widely explored due to huge professional barriers. Herein, the characteristics of the tumor immune microenvironment and the mechanisms of ICD are firstly systematically summarized. Subsequently, the therapeutic mechanism of SDT is fully summarized, and the advantages and limitations of SDT are discussed. The representative advances of SDT‐based nanomedicines for cancer immunotherapy are further highlighted. Finally, the application prospects and challenges of SDT‐based immunotherapy in future clinical translation are discussed.

## Graphical abstract

Biological characteristics of the tumor immune microenvironment (TIM) and the mechanisms of immunogenic cell death (ICD) are described. Anticancer mechanisms of sonodynamic therpy (SDT) and its advantages and limitations are summarized. Represantative advances of SDT based nanomedicines for cancer immunotherapy are highlighted. Urgent challenges and clinical application prospects of SDT based immunotherapy are discussed.

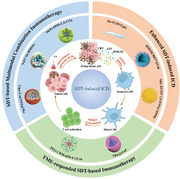

## 1 Introduction

**Figure 1:**
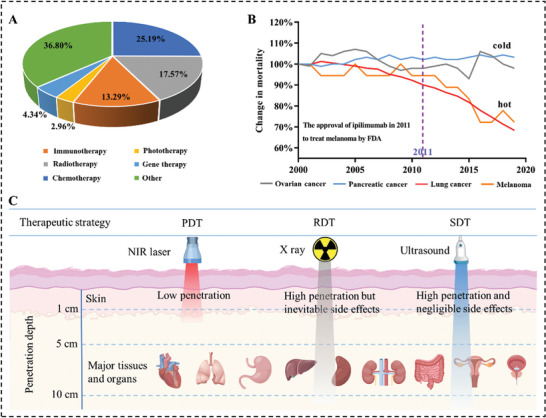
A) Distribution of various cancer therapies in cancer clinical trials in the United States from 2020 to date. B) Changes in mortality of different types of tumors from 2000 to 2019, two “cold” tumors (ovarian cancer and pancreatic cancer) and two “hot” tumors (lung cancer and melanoma). C) Comparison of different therapeutics in tissue penetration. NIR‐based PDT laser has poor tissue penetration (around 1–5 cm) which is limited to superficial cancers. X ray has high tissue penetration (limitless) but inevitable side effects, which limit the biomedical applications of RDT. US not only has high tissue penetration (>10 cm) but also has negligible side effects, which makes SDT favorable for deep tumors.

Immunotherapy is a class of methods to treat cancer by activating the immune system, mainly covering immune checkpoint blockade (ICB) therapy,^[^ ^1^ ^]^ cytokine therapy,^[^ ^2^ ^]^ adoptive cell therapy,^[^ ^3^ ^]^ and cancer vaccines.^[^ ^4^ ^]^ In the past decade, immunotherapy has profoundly changed the treatment paradigm of cancer as an emerging treatment method.^[^ ^5^ ^]^ ICB therapy has demonstrated impressive therapeutic effects in melanoma. In 2011, the ipilimumab, the first antibody blocking an immune checkpoint, was approved by the FDA for the treatment of melanoma, marked the beginning of the cancer immunotherapy revolution.^[^ ^6^ ^]^ The immune responses and self‐tolerance of T cell are precisely regulated by several evolutionarily conserved T cell activating factors, such as cytotoxic T lymphocyte‐associated antigen 4 (CTLA‐4) and programmed death receptor 1 (PD‐1). James P. Allison and Tasuku Honjo won the 2018 Nobel Prize in Physiology or Medicine for their successful development of inhibitors of CTLA4 and PD‐1 to treat a variety of intractable cancers. Currently, PD‐1 and programmed death‐ligand 1 (PD‐L1) antibodies have become the most widely used immunotherapy drugs to treat several different types of tumors, including non‐small cell lung cancer,^[^ ^7^ ^]^ head‐and‐neck squamous cell carcinoma,^[^ ^8^ ^]^ and advanced cutaneous squamous cell carcinoma.^[^ ^9^ ^]^ The five‐year survival rate of these tumors has improved significantly in the past decade thanks to the wide application of immunotherapy. Encouraged by these excellent outcomes, numerous of immunotherapy‐based clinical trials have been extensively explored for the treatment of many other tumor types recently, accounting for ≈13.29% of all oncology clinical trials in the United States from 2020 to now (**Figure** **1**A).^[^ ^10^ ^]^ However, immunotherapy has only very limited success and has poor efficacy against many types of cancer, such as ovarian cancer,^[^ ^11^ ^]^ pancreatic cancer,^[^ ^12^ ^]^ and glioblastomas.^[^ ^13^ ^]^ These cancers mainly have a “cold” tumor immune microenvironment (TIM) with little infiltration of inflammatory cells (including T cells, macrophages, etc.). As a result, immunotherapy is greatly compromised in these cold cancers than these cancers with a “hot” immune microenvironment, such as lung cancer and melanoma (Figure 1B).^[^ ^14^ ^]^

It is an effective strategy to improve the effect of immunotherapy by shifting the TIM from cold to hot.^[^ ^15^ ^]^ Cancer immunogenic cell death (ICD) can greatly promote inflammatory cell infiltration and turn the cold TIM into hot in solid tumors by some emerging ROS‐mediated cancer therapy, like radiodynamic therapy (RDT),^[^ ^16^ ^]^ photodynamic therapy (PDT),^[^ ^17^ ^]^ and sonodynamic therapy (SDT).^[^ ^18^ ^]^ These treatments have been widely developed for the treatment of cancer because of many significant merits over traditional treatments (chemotherapy and operation treatment),^[^ ^19^ ^]^ such as high spatiotemporally selective, low‐invasive, and strong therapeutic effect. Different from RDT and PDT, the antitumor effects of SDT are multiple. On the one hand, ultrasound (US) can generate sonoluminescence through the cavitation effect, activating sonosensitizers to generate electrons, which can react with surrounding oxygen (O_2_) to produce cytotoxic singlet oxygen (^1^O_2_), inducing apoptosis of cancer cells. On the other hand, shock waves and shear stress generated in the process of acoustic cavitation can also cause mechanical damage to cancer cells. Among these treatments, PDT has been approved by the FDA for the treatment of cancer due to the advantages of high efficiency and low side effects,^[^ ^20^ ^]^ but it is only more suitable for superficial tumor treatment due to the poor tissue penetration depth of near‐infrared (NIR) light (around 1–5 cm).^[^ ^21^ ^]^ RDT has deep penetrating ability, but ionizing radiation can greatly damage normal tissues.^[^ ^22^ ^]^ By comparison, SDT is the only therapy with the advantages of low side effects as well as deep tissue penetration (>10 cm) (Figure 1C). Therefore, SDT has greater clinical translation potential and value than PDT and RDT. In 2009, it was first reported that SDT was conducted to treat advanced refractory breast cancer patients who failed to respond to conventional treatments.^[^ ^23^ ^]^ Henceforth, SDT has shown encouraging clinical benefits by combining with other treatments, such as PDT^[^ ^24^ ^]^ and immunotherapy.^[^ ^25^ ^]^ Notably, the significant acute inflammatory responses and the activation of host's antitumor immunity were observed in these cases after treatments, which not only eliminated primary tumors but destroyed metastatic tumors. These clinical case reports proved the potential of SDT‐based immunotherapy in enhancing therapeutic effect and improving the patients’ tolerance. However, SDT‐induced antitumor immunity is substantially limited by hypoxic tumor microenvironment (TME)^[^ ^26^ ^]^ and insufficient ROS yield.^[^ ^27^ ^]^ Excitingly, the SDT‐based nanoplatforms adopted multiple enhancement strategies to improve antitumor immune response efficiency, such as improving tumor targeting, enhancing ROS yield, relieving tumor hypoxia, combining SDT with chemotherapy, chemodynamic therapy (CDT), PDT and/or photothermal therapy (PTT) to maximize the treatment efficiency.

**Figure 2:**
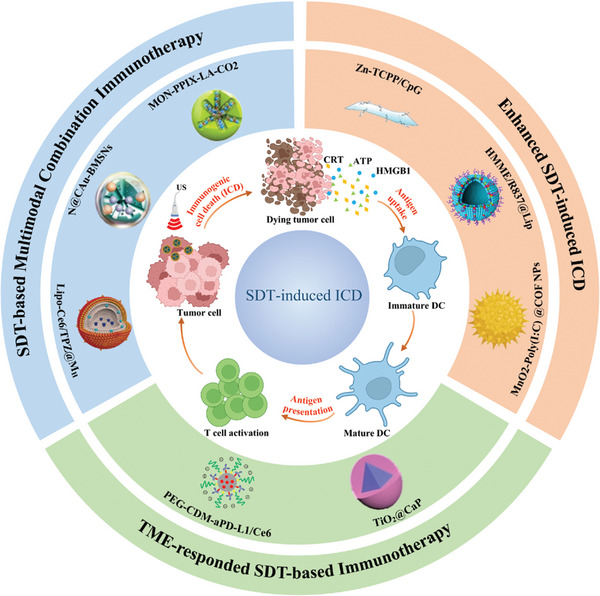
The scope and focus of this article. SDT can induce ICD and the release of antigens and DAMPs. DAMPs and antigens promote the maturation of DCs and activate T cells to induce antitumor immune responses. Recent researches of SDT‐based immunotherapy are summarized into three aspects: Enhanced SDT‐induced ICD, TME‐responded SDT‐based immunotherapy, and SDT‐based multimodal combination immunotherapy.

Currently, many emerging SDT‐induced ICD nanomedicines have been developed to treat cancer, and these nanomedicines can effectively clear not only large primary tumors but also metastases. However, this field has high specialization barriers and involves multiple interdisciplinary fields such as immunology, molecular biology, chemistry, and materials. Therefore, SDT‐based immunotherapy nanoplatforms are far from being widely developed despite their great promise. Herein, for the first time, we comprehensively review the latest progress of SDT‐based immunotherapy with nanomedicines for cancer treatment. First, we systematically summarize the characteristics of the TIM and the mechanisms of ICD. Subsequently, the therapeutic mechanisms of SDT are fully summarized, and the advantages and limitations of SDT are discussed. The representative researches of SDT‐based cancer immunotherapy are further highlighted, including the following three aspects, that is, adjuvant‐enhanced SDT‐based immunotherapy, TME‐responded SDT‐based immunotherapy, and SDT‐based multimodal combination immunotherapy (**Figure** **2**). Finally, the prospects and challenges of SDT‐based immunotherapy with nanomedicines are discussed in future clinical translation.

## 2 TIM and ICD

**Figure 3:**
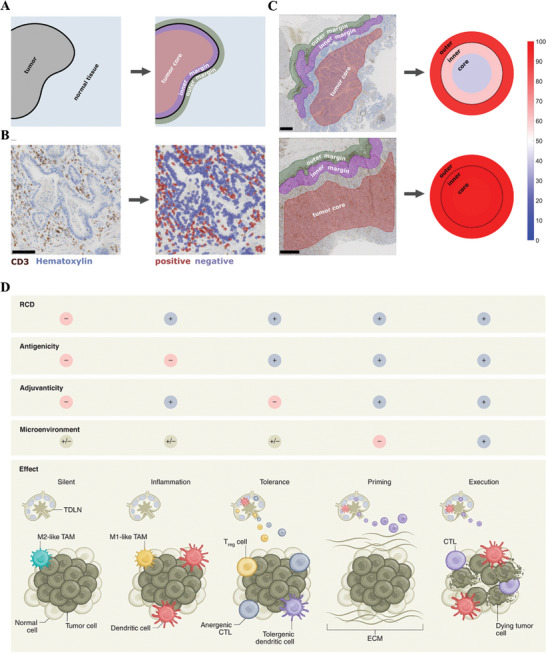
A) Tumors can be delineated into three compartments: outer 500 µm invasive margin, inner 500 µm invasive margin, and tumor core. B) Automatic cell detection of CD3‐stained gastric carcinoma slide. C) Creating a “target plot” where the three color corresponds to the percentile‐normalized cell density. The upper sample is an immune‐excluded phenotype while the lower sample is an inflamed phenotype. Adapted with permission.^[^ ^29^ ^]^ Copyright2021, Elife Sciences Publ Ltd. D) Schematic diagram of the different effects of RCD under distinct conditions to initiate an adaptive immune response. Three key parameters are required for RCD to initiate an adaptive immune response, including antigenicity, adjuvanticity, and a permissive microenvironment. In the absence of antigens, RCD can only drive inflammatory responses. In the presence of antigenicity and sufficient adjuvanticity, RCD can initiate adaptive immune responses. Conversely, the presentation of antigenic determinants to T cells actively promotes tolerance in the setting of poor adjuvanticity. Finally, the TME dictated whether activated T cells can access tumor lesions to mediate effector functions and establish a memory response. Adapted with permission.^[^ ^37^ ^]^ Copyright 2021, Nature portfolio.

TIM lays an important role in regulating tumor growth, invasion and metastasis.^[^ ^28^ ^]^ The characteristics of TME include many aspects, such as slightly acid, high concentration of hydrogen peroxide (H_2_O_2_), hypoxia, and immune microenvironment. In particular, the TIM has a decisive impact on the efficacy of cancer immunotherapy. The TIM is divided into three typical types according to the degree and distribution of inflammatory cell infiltration in the tumor.^[^ ^29^ ^]^ First, the hot TME, that is, inflammatory cells are distributed around and inside the tumor. Second, exclusion TME, that is, inflammatory cells are only distributed in the tumor periphery, and not distributed in the interior. The third type, the cold TME, has few or no inflammatory cells distributed inside or periphery of the tumor (**Figure** **3**A–C). In fact, most types of tumors have cold immune microenvironments, such as exclusion and cold types. For instance, ICB has an excellent effect on only a small fraction of cancer patients with a hot TME (about 10%–35% of the total),^[^ ^30^ ^]^ and is ineffective or moderately effective in most patients with immune‐excluded and cold immune microenvironments.^[^ ^31^ ^]^ In the cold TME, abundant immunosuppressive cells accumulate in the tumor, like regulatory T cells (Tregs),^[^ ^31^ ^]^ M2‐like tumor‐associated macrophages (M2‐like TAMs),^[^ ^32^ ^]^ and myeloid‐derived suppressor cells (MDSCs).^[^ ^33^ ^]^ Many kinds of immunosuppressive proteins, such as PD‐L1 and CD47, are highly expressed on the surface of tumor cells to inhibit the immune activity of various immune cells by binding to their specific surface receptors (such as PD‐1 and SIRP‐*α*).^[^ ^34^ ^]^ Moreover, tumor‐associated fibroblasts (TAFs) are a huge barrier to tumor immunotherapy. The thick stroma is formed by secretions of TAFs (like collagen, fibrin, and extracellular matrix form thick stroma), which separate tumor nests from the immune system and provide the stable structure of the TME.^[^ ^35^ ^]^ In addition, the hypoxic TME plays an important role in hindering tumor immunotherapy. Hypoxic microenvironment promotes the expression of immunosuppressive factors like PD‐1, PD‐L1, and CD86 (the immune checkpoint CTLA‐4 ligand) via hypoxia‐inducible factor‐*α* (HIF‐*α*).^[^ ^36^ ^]^

**Figure 4:**
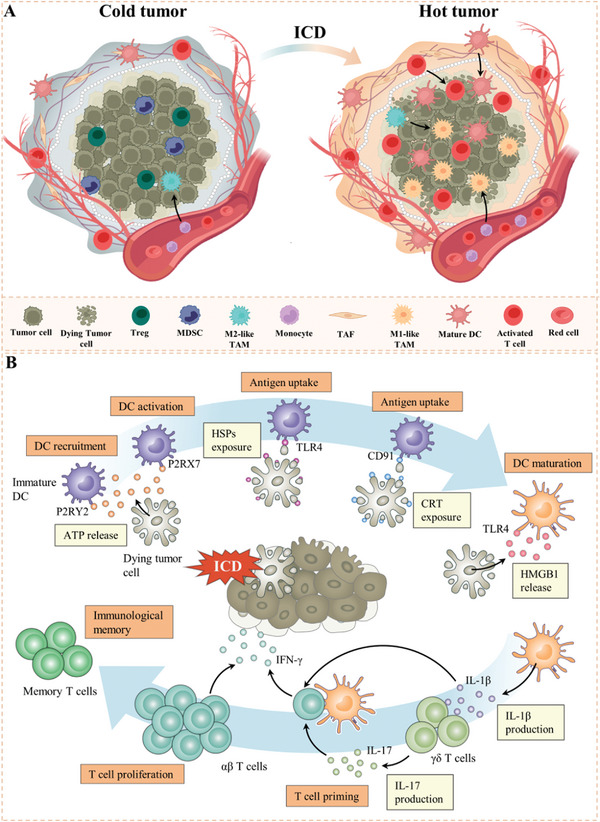
A) The characteristics of cold tumors and hot tumors. Cold tumors have nonimmunogenic microenvironment, including immunosuppressive cells Tregs, MDSCs, and M2‐like TAM. ICD makes cold tumor toward hot, which form a highly immunogenic microenvironment with infiltrated activated T cells, mature DCs, and M1‐like TAM. B) Schematic illustration of the process of ICD. In response to ICD inducers, malignant cells expose CRT and HSPs on surface, secrete ATP, and release HMGB1. Upon binding to cognate receptors on the surface of myeloid or lymphoid cells, these DAMPs favor the uptake of cell corpses and debris thereof by APCs, including DCs in the context of robust immunostimulatory signals, which eventually leads to the priming of an adaptive immune response involving both *αβ* and *γδ* T cells.

Regulatory cell death (RCD) is a controllable form of cell death, which is mediated by specific signaling cascades and molecular mechanisms.^[^ ^38^ ^]^ RCD covers various forms of cell death, such as apoptosis, autophagy, pyroptosis, ferroptosis, and necroptosis.^[^ ^39^ ^]^ As a specific variant of RCD, ICD is driven by intracellular stress responses and activating adaptive immune responses in immunocompetent hosts. Not all RCD can initiate an adaptive immune response. For example, when only one of the adjuvanticity or antigenicity is met, RCD can only drive the inflammatory response or promote tolerance, and the initiation of adaptive immunity only when the two conditions are met at the same time. Intriguingly, adaptive immune responses driven by ICD can only be executed if the antigenicity, adjuvanticity and permissive microenvironmental conditions exist simultaneously (Figure 3D).^[^ ^40^ ^]^ In particular, the tumor cells themselves are antigen‐rich and immunogenic‐strong despite the cold TME of many types of cancer.^[^ ^37^ ^]^ For example, ovarian cancer cells express many cancer antigens, such as epithelial cell adhesion molecule, cancer antigen 125, *α*‐folate receptor, etc.^[^ ^41^ ^]^ Generally, normal cells death only causes inflammation without an adaptive immune response because normal cells are immunogenic‐weak, and inflammatory cells (such as macrophages) will quickly recognize, engulf and eliminate these dead cells. Conversely, ICD of cancer cells not only causes inflammation but also generates antigen‐specific immune responses that are executed by cytotoxic T lymphocytes (CTLs) and trigger immunological memory,^[^ ^37^ ^]^ which has shown great potential for converting immunologically cold tumors to hot (**Figure** **4**A).^[^ ^42^ ^]^ In the course of ICD, plentiful danger‐associated molecular patterns (DAMPs) from dying tumor cells can act as endogenous adjuvant signals to stimulate inflammatory cell infiltration and drive antigen‐presenting cells (APCs) maturation,^[^ ^43^ ^]^ such as ATP, calreticulin (CRT), heat shock proteins (HSPs), and high mobility group box 1 (HMGB1) (**Table** **1**). For instance, extracellular ATP released by dying cancer cells act as a “find me” signal to mediate the recruitment, activation, and inflammatory effects of APCs by binding to P2RY2 or P2RX7 purinergic receptors on APCs, especially dendritic cells (DCs).^[^ ^44^ ^]^ In addition, ATP stimulate the secretion of interleukin‐1*β* (IL‐1*β*) by activating the caspase‐1‐dependent NLRP3 inflammasome, and induces CTLs and *γδ* T cells to mediate antitumor immune response by IL‐17 and interferon (IFN).^[^ ^45^ ^]^ The surface‐exposed calreticulin (Ecto‐CRT) is activated by a ROS‐based endoplasmic reticulum (ER) stress response that involve the phosphorylation of the eukaryotic translation initiation factor 2*α* (eIF2*α*) by the protein kinase‐like endoplasmic reticulum kinase (PERK).^[^ ^43^ ^]^ The surface‐exposed calreticulin is the key to triggering antitumor immune responses. Ecto‐CRT promotes the recruitment of APCs, antigen presentation, release of pro‐inflammatory cytokines, and activation of T helper cell 17 (Th17) cells through the recognition, phagocytosis, and binding of CD91‐expressing cells, and finally initiates the antitumor immune response.^[^ ^46^ ^]^ The surface‐exposed heat shock proteins (such as HSP70 and HSP90) are also an important DAMPs that act as an “eat‐me” signal, which promote tumor‐associated antigen (TAA) uptake and presentation by binding to Toll‐like receptor 4 (TLR4) on immature DCs.^[^ ^47^ ^]^ HMGB1 also promotes DCs activation and migration and stimulates the release of proinflammatory cytokines by binding to multiple pattern recognition receptors (PRRs), such as TLR4 and receptor for advanced glycation end products (RAGE) (Figure 4B).^[^ ^48^ ^]^

**Table 1:** DAMPs in ICD

DAMPs	Category	Mechanism of release/exposure	Receptors	Contribution to ICD	Ref.
ATP	“Find me”	Exocytosis; passive release	P2RX7 P2RY2	Favors the recruitment of APCs and their activations by activating NLRP3 inflammasome	[44]
CRT	“Eat me”	Exocytosis;phosphatidyl‐serine based exposure fromcytosol; anterograde transport of vesicles from the ER to the Golgi apparatus	CD91	Promotes the recruitment of APCs, antigen presentation, release of proinflammatory cytokines, and activation of Th17 cells	[46]
HSPs	“Eat me”	Released from necrotic cells, especially when they have been induced by prior stress	TLR4	Stimulates the uptake of dead cell‐associated antigens	[47]
HMGB1	Others	Released from the nucleus upon its acetylation and/or activation of poly(ADP‐ribose)‐polymerase‐1	TLR4 RAGE	Stimulates the release of proinflammatory cytokines, activates macrophages, and induces DC maturation	[48]

The TME also has a strong impact on immunotherapy triggered by ICD. For example, the highly expressed immune checkpoints in the TME still make CTLs lose their immunotherapeutic effect although ICD can increase the infiltration of CTLs. Therefore, ICD combined with ICB restored the efficient antitumor role of CTLs.^[^ ^49^ ^]^ In addition, not all treatments can induce ICD of cancer cells. In general, only these DAMPs were involved in cell surface changes to induce ICD of cancer. ROS led to ER turnover by mediating ER stress, which induces surface changes in cancer cells to expose cancer cell antigens.^[^ ^43^, ^50^ ^]^ Therefore, ROS based treatments can efficiently induce ICD, such as PDT, RDT, and SDT.

## 3 SDT for Cancer Immunotherapy

**Figure 5:**
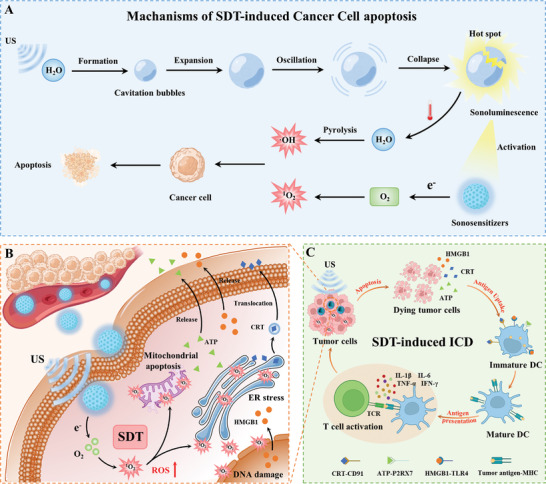
A) Schematic illustration of the anticancer mechanisms of SDT. US irradiation caused a cavitation effect to generate cavitation bubbles by repeatedly compressing H_2_O. An instantaneous hot spot with high temperature and high pressure is formed to generate self‐luminescence from ultraviolet to visible light, that is, sonoluminescence, after the expansion, oscillation, and collapse of cavitation bubbles. Subsequently, the sonosensitizers activated by sonoluminescence convert electrons to O_2_ to produce cytotoxic ^1^O_2_, which induces the apoptosis of cancer cells. At the same time, the localized high temperature generated from hot spot can pyrolyze H_2_O to cytotoxic ·OH to further inducing cancer cell apoptosis. B) Schematic illustration of SDT‐induced ICD. Under US radiation, sonosensitizers convert O_2_ to ^1^O_2_ to induce DNA damage, ER stress, and mitochondrial apoptosis. Subsequently, HMGB1 and ATP are released from dying cancer cells, while CRT is translocated to the cell membrane surface via the ER‐Golgi transport pathway. C) Schematic illustration of antitumor immune responses triggered by SDT‐induced ICD. Sonosensitizers induce cancer cell apoptosis by ^1^O_2_ under US irradiation, and then dying cancer cells promote DC maturation through surface‐exposed CRT, released ATP and HMGB1. Subsequently, mature DC activate T cells to CTLs, and antitumor immune responses were triggered finally.

The antitumor effect of SDT involves multiple physical‐chemical processes that take place during acoustic cavitation induced by US irradiation.^[^ ^51^ ^]^ Generally, acoustic cavitation can be distinguished into two types: stable cavitation and inertial cavitation. Similarly, the cytotoxic effects of SDT mainly include two aspects, that is, mechanical damage from cavitation effect and chemical damage from ROS. During stable cavitation, gas pockets formed and oscillated around an equilibrium radius through acoustic compression and decompression cycles, which generated fluid streaming and shear stresses to destroy cancer cells.^[^ ^52^ ^]^ In addition, the shock waves generated from collapsed bubbles during inertial cavitation have been reported to mechanically damage cancer cells.^[^ ^52^, ^53^ ^]^ Most importantly, inertial cavitation can generate cavitation bubbles by rhythmically compressing H_2_O. With the expansion and final collapse of cavitation bubbles, an instantaneous hot spot with high temperature (>5000 K) and high pressure (>800 atm) was formed,^[^ ^52b^ ^]^ which generated self‐luminescence from ultraviolet to visible light, that is, sonoluminescence.^[^ ^54^ ^]^ Subsequently, sonosensitizers were activated by sonoluminescence, and finally transferred electrons to O_2_ to generate cytotoxic ^1^O_2_ (**Figure** **5**A). At the same time, the localized high temperature from collapsed bubbles can pyrolyze H_2_O to hydroxyl radical (·OH). Based on these, SDT can effectively induce ICD to activate antitumor immune responses via ROS‐mediated cancer cell apoptosis pathway (Figure 5B,C). The mechanism of SDT and PDT were similar, and both involved light to generate ROS. Therefore, most of the sonosensitizers were derived from photosensitizers (**Table** **2**).^[^ ^55^ ^]^ Notably, SDT was more attractive in inducing ICD in deep‐lying tissues malignant tumor thanks to the excellent tissue penetration of US. Importantly, the low‐intensity US exposure had negligible side effects and the cytotoxicity was only caused by the transient spatiotemporal overlap of the sonosensitizers and US.^[^ ^56^ ^]^ In addition, the therapeutic effect of SDT can be further optimized by focusing on the tumor site and adjusting the US frequency, intensity, and irradiation time.

**Table 2:** Overview of common nanosensitizers for SDT

Categories	Sonosensitizers	Mechanism	Power	Biologic model	Ref
Porphyrins	Protoporphyrin	ROS	1 MHz, 0.12 W cm^−2^	OSCC	[64]
	Hematoporphyrin	ROS	1.8 MHz, 3 W cm^−2^	S180 cell	[65]
	Photofrin	ROS	1.92 MHz, 3 W cm^−2^	Colon 26 carcinoma	[66]
	Hematoporphyrin monomethyl ether	ROS	1 MHz, 1.5 W cm^−2^	A‐253 cell	[67]
	Chlorin e6	ROS	1 MHz, 0.36 W cm^−2^	4T1 cell	[68]
Phthalocyanines	Aluminum phthalocyanine	ROS	3 MHz, 3 W cm^−2^	Colon‐26 cell	[69]
	ZnPcS_2_P_2_	ROS	1 MHz, 0.5 W cm^−2^	U251 glioma	[70]
Xanthenes	Rose bengal	ROS	1 MHz, 1.5 W cm^−2^	RIF‐1 cell	[71]
	Rose bengal derivatives	ROS	1 MHz, 2 W cm^−2^	HepG2	[72]
	Erythrosine B	ROS	1.2 MHz, 3.1 W cm^−2^	U937 cell	[73]
Anti‐inflammatory drugs	Levofloxacin	ROS	40 kHz, 1 W cm^−2^	BSA	[74]
	Piroxicam	ROS	2 MHz, 10 W cm^−2^	S180 cell	[75]
	Quinolone antibiotics	ROS	2 MHz, 2 W cm^−2^	S180 cell	[76]
Antitumor drugs	Adriamycin	ROS chemotherapy	1.93 MHz, 5 W cm^−2^	S180 cell	[77]
Phenothiazine compounds	Methylene blue	ROS	0.46 MHz, 1.7 W cm^−2^	HO‐8910 cell	[78]
Natural products	Artesunate	ROS	3 MHz, 3 W cm^−2^	EMT‐6 mammary tumor cell	[79]
	Hydroxysafflor yellow A	ROS	1 MHz, 0.4 W cm^−2^	THP‐1 macrophages	[80]
Other organic molecules	Aminolevulinic acid	ROS	1.1 MHz, 1 W cm^−2^	HUVECs	[81]
	Indocyanine green	ROS	1 MHz, 1 W cm^−2^	MH7A cell	[82]
Inorganic sonosensitizer	TiO_2_ NPs	ROS	1 MHz, 0.5 W cm^−2^	Panc02 cell	[83]
	MnWOX NPs	ROS	40 kHz, 3 W cm^−2^	4T1 cell	[84]
	Au‐MnO NPs	ROS	1 MHz, 2 W cm^−2^	MCF‐7 cell 97H cell	[85]

However, SDT‐induced ICD were limited by these traditional sonosensitizer's ROS yield and bioavailability in vivo. Generally, traditional sonosensitizers were small molecule organic sonosensitizers (e.g., porphyrins and their derivatives,^[^ ^57^ ^]^ phthalocyanines,^[^ ^58^ ^]^ etc.). In addition, some inorganic substances such as TiO_2_ were adopted as sonosensitizers.^[^ ^59^ ^]^ However, it was difficult for these sonosensitizers to reach the tumor site through the blood circulatory system. The efficacy of sonosensitizers was also limited by the tumor hypoxic microenvironment. Moreover, the high concentration of glutathione (GSH) limited the effect of SDT in tumor although the O_2_‐independent sonosensitizers improved the efficiency of ROS generation to a certain extent.

Recently, numerous efforts have been devoted to further improve the sonosensitization efficiency, bioavailability, and tumor targeting of sonosensitizers for SDT, as well as SDT‐based multimodal combination therapy.^[^ ^18^, ^60^ ^]^ Specially, sonosensitizers have been developed to enhance the therapeutic effect of SDT‐based immunotherapy with the rapid development of nanotechnology and nanoscience. Well‐designed sonosensitizers greatly improved the SDT‐induced ROS yield by enhancing acoustic cavitation effect^[^ ^61^ ^]^ or alleviating tumor hypoxia.^[^ ^62^ ^]^ Modifiable nanocarriers also significantly increased the bioavailability and tumor targeting of sonosensitizers by prolonging the blood circulation time and the accumulation in tumor tissues.^[^ ^63^ ^]^ Recently, many emerging SDT‐based nanoplatforms were developed to improve the effect of immunotherapy, and demonstrated amazing antitumor effect by inducing ICD. These SDT‐based nanoplatforms focus on the following three parts: 1) SDT‐based nanoplatforms further amplify ICD effect to reverse the cold TIM by enhanced SDT; 2) SDT‐based nanoplatforms improve the immune‐suppressing microenvironment by specifically responding to TME; and 3) SDT‐based multifunctional therapeutic platforms synergistically treat cancer by combining multiple therapeutic modalities (**Table** **3**).

## 4 Enhanced SDT‐Induced ICD

In 2018, Zhang et al. first developed SDT as an adjunctive immunotherapy approach to treat cancer with HiPorfin (a commercial sonosensitizer).^[^ ^86^ ^]^ SDT not only induced the transformation of immunosuppressive Th2 cells, TAM cells into TH1 and M1 macrophages, but also promoted adaptive immune cells with high infiltration of CD4 and CD8 T cells by intratumoral injection of HiPorfin and subsequent US stimulation. However, conventional sonosensitizers have low bioavailability and unsatisfactory therapeutic efficiency, which require prolonged sonication and multiple intertumoral injections to obtain sufficient immunotherapeutic efficiency.^[^ ^87^ ^]^ Recently, some emerging nanomedicine‐based strategies had been developed to improve the therapeutic efficiency of sonosensitizers,^[^ ^88^ ^]^ mainly including integrating immune adjuvants and enhancing ROS yield by chemiluminescence resonance energy transfer (CRET).

### 4.1 Adjuvant‐Enhanced SDT‐Based Immunotherapy

As nonspecific immune enhancers, immune adjuvants have been focusing on modulating DCs function to improve cancer immunotherapy,^[^ ^89^ ^]^ because they can further amplify the immunotherapeutic effects by eliciting a strong inflammatory response. Therefore, the combination of SDT and immune adjuvants are an powerful therapeutic intervention for enhancing antitumor immune responses.^[^ ^90^ ^]^ Currently, there are two main types of immune adjuvants for SDT‐based cancer immunotherapy, namely toll‐like receptor (TLR) agonists^[^ ^90^, ^91^ ^]^ and the activator of cyclic GMP‐AMP synthase (cGAS)‐stimulator of interferon genes (STING) pathway.^[^ ^92^ ^]^

#### 4.1.1 TLR Agonists for SDT‐Based Immunotherapy

Cytosine‐phosphorothioate‐guanine (CpG), as an analog of microbial DNA, can be recognized as pathogen‐associated molecular patterns (PAMPs) by immune cells with TLR9.^[^ ^93^ ^]^ Very recently, Zhu et al. developed a 2D nanosonosensitizer (Zn‐TCPP) loaded with CpG (a potent TLR9 agonist) to enhance SDT‐based immunotherapy.^[^ ^91a^ ^]^ The 2D Zn‐TCPP was prepared by self‐assembly of tetrakis(4‐carboxyphenyl) porphyrin (TCPP) and Zn^2+^. The 2D Zn‐TCPP, as a metal–organic framework (MOF), not only avoided the reduction of ROS yield caused by stacking of TCPP, but also efficiently loaded CpG thanks to their porous nature. As a result, the 2D Zn‐TCPP exhibited high SDT efficacy to induce cancer cell apoptosis due to efficient electron conduction and reduced self‐quenching of TCPP. Importantly, TAAs derived from SDT together with CpG performed whole‐tumor‐cell vaccines to trigger more robust ICD as verified by CRT exposure and ATP release, thereby inducing intense antitumor immune responses to inhibit distant abscopal tumors, as well as triggering strong immunological memory to prevent tumor recurrence.

Similarly, Chen et al. developed a nanosonosensitizer (HMME/R837@Lip) co‐loaded with hematoporphyrin monomethyl ether (HMME, a sonosensitizer) and R837 (as a TLR7 agonist) for the combination of immune adjuvant, SDT, and anti‐PD‐L1 immunotherapy.^[^ ^91b^ ^]^ The HMME/R837@Lip was constructed by coencapsulating the HMME and the immune adjuvant R837 (TLR7 agonist) into an FDA‐approved liposome. Under US irradiation, HMME in HMME/R837@Lip effectively generated ^1^O_2_ to induce elicit ICD to facilitate DCs maturation and cytokine secretion via synergistic vaccine‐like functions of SDT‐generated TAAs and R837. Moreover, the antitumor immune responses further enhanced after combination with anti‐PD‐L1 immunotherapy, and the tumor growth both in primary orthotopic and mimic distant tumors were inhibited significantly with long‐term immunological memory to prevent tumor recurrence.

#### 4.1.2 cGAS‐STING Pathway Activator for SDT‐Based Immunotherapy

**Figure 6:**
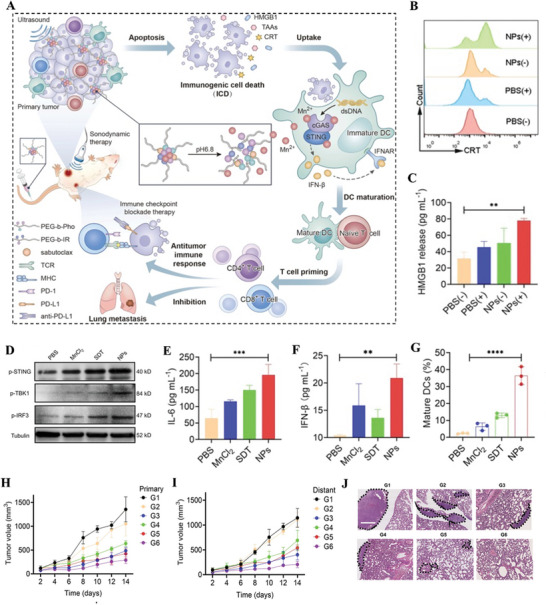
A) Schematic representation of phenolic nanoadjuvant for promoting significant maturation of DCs and enhancing PD‐L1 checkpoint blockade immunotherapy. B) Flow cytometric analyses of CRT expression in 4T1 cells after different treatments. C) ELISA analysis of HMGB1 release from 4T1 cells after various treatments for 24 h. D) After different treatments with PBS, MnCl_2_, SDT or PIMS NPs, 4T1 cells were incubated with BMDCs and followed by western blot analysis of the activation of cGAS STING pathway. E,F) The secretion of IL‐6 and IFN‐*β* in mature DCs suspensions evaluated by ELISA analysis. G) Quantitative analyses of mature DCs. H,I) Tumor volume growth curves for primary tumors and distant tumors, respectively, after different treatments. J) H&E staining of lung metastasis, the tumor area in lung is marked with a black dashed line. Adapted with permission.^[^ ^92^ ^]^ Copyright 2022, Elsevier Sci Ltd.

The cGAS‐STING pathway, as an important member of the innate immune system, detect the presence of cytoplasmic DNA to inhibit tumor development by promoting DCs maturation.^[^ ^94^ ^]^ Very recently, Tian and co‐workers developed a phenolic nanoadjuvant (PEG‐IR‐Mn^2+^‐sabutoclax nanoparticles, PIMS NPs) via SDT‐induced ICD, the activation of cGAS‐STING pathway and PD‐L1 checkpoint blockade immunotherapy for enhancing antitumor immunity (**Figure** **6**A).^[^ ^92^ ^]^ The PIMS NPs were constructed by self‐assembly of the sonosensitizer polymer (PEGb‐IR), GSH inhibitor sabutoclax, Mn^2+^, and acid‐sensitive phenolic polymer (PEGb‐Pho) based on metal–phenolic coordination. In the multifunctional PIMS NPs, the sonosensitizer PEGb‐IR triggered ROS generation under US irradiation, while sabutoclax prevented ROS reduction by reducing GSH levels to further amplify SDT effect to induce severe cancer cell death. Therefore, this enhanced SDT effect induced robust ICD, which was verified by observation of massive CRT exposure and HMGB1 release (Figure 6B,C). Meanwhile, the PIMS NPs activated the cGAS‐STING pathway through sustained release of Mn^2+^ to recognize tumor‐derived DNA in DCs, and stimulated the release of IFN‐*β* to significantly foster the maturation of DCs (Figure 6D–G). The PIMS NPs not only effectively inhibited orthotopic tumor growth by SDT, but also greatly sensitized cancer cells to anti‐PD‐L1 immunotherapy via the synergistic effect of Mn^2+^ and ICD, substantially delayed distal tumor growth and inhibited lung metastasis in both orthotopic and bilateral 4T1 tumor models (Figure 6H–J).

### 4.2 CRET‐Enhanced SDT‐Based Immunotherapy

Chemiluminescence have been widely used in the detection of cancer and acute liver injury (AKI) because of its high sensitivity.^[^ ^95^ ^]^ During some specific chemical reactions, the newly generated substances in the excited state returned to the ground state and emitted luminescence, that was, chemiluminescence.^[^ ^96^ ^]^ The energy of chemiluminescence was efficiently transferred to the sonosensitizer to generate ROS through CRET when the spectrum of chemiluminescence overlapped with the absorption spectrum of the sonosensitizer and the distance between the high‐energy state species and the sonosensitizer was close enough.^[^ ^97^ ^]^ Therefore, it was an efficient strategy to achieve CRET‐enhanced SDT by coloading of chemiluminescent precursors and sonosensitizers into a same nanocarrier. Very recently, Jueun et al adopted CRET based nanoparticles (iCRET NPs) to further enhance the ROS yield for SDT‐induced ICD.^[^ ^98^ ^]^ The iCRET NPs were prepared from polyethylene glycol (PEG), verteporfin (VPF, a hydrophobic sonosensitizer), and butylene oxalate by emulsion method. iCRET NPs were efficiently enriched to tumor sites through the ERP effect. The high levels of H_2_O_2_ oxidized butylene oxalate to generate CO_2_ bubbles, accompanied by chemiluminescence that transferred energy to verteporfin for enhanced ROS yield. Based on this ingenious reaction, iCRET NPs double enhanced the SDT effect through CO_2_ bubbles and CRET. As result, iCRET NPs significantly induced ICD in CT26 tumor‐bearing mice. Under US irradiation, both primary tumors and lung metastases were significantly inhibited in CT26 tumor‐bearing mice after iCRET NPs treatment.

## 5 TME‐Responded SDT‐Based Immunotherapy

The microenvironment of tumor sites is significantly different from that of normal tissues, such as a slightly acidic environment.^[^ ^99^ ^]^ The unique TME can provide powerful active targeting for specially designed nanomedicines to greatly increase the effect of SDT‐induced ICD and reduce side effects. Some pH‐responsive nanosensitizers can actively respond to the acidic TME to intelligently turn on SDT and greatly improve the accuracy of cancer treatment.^[^ ^100^ ^]^

**Figure 7:**
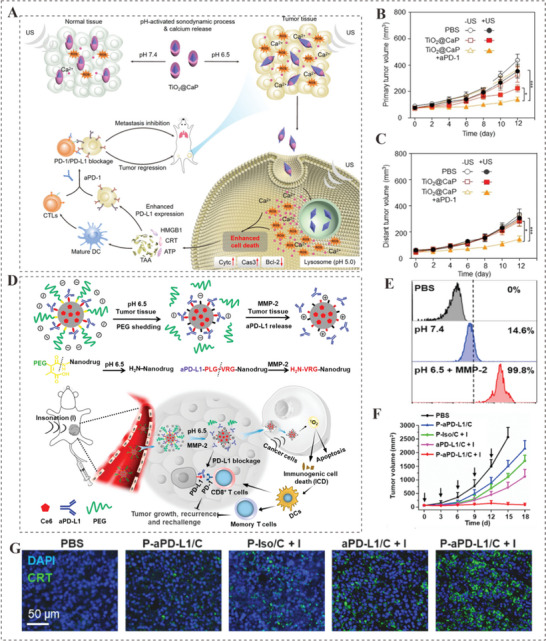
A) Schematic representation of the transformable TiO_2_@CaP in different conditions and the mechanism on boosting sono‐immune therapeutic efficacy in tumor. B,C) Growth curves of primary and distant tumors in 4T1 tumor‐bearing mice after various treatments. Adapted with permission.^[^ ^100a^ ^]^ Copyright 2021, Wiley‐VCH. D) Diagram of the pH and MMP‐2 dual‐sensitive PEG‐CDM‐aPD‐L1/Ce6 for tumor‐targeting immune‐sonodynamic therapy of cancer. E) Cellular uptake of nanodrug measured by flow cytometry. F) Tumor growth of animals receiving different treatments. G) Immunofluorescent staining of CRT expression of cancer cells in different treatment groups. Adapted with permission.^[^ ^100b^ ^]^ Copyright 2021, Elsevier Sci Ltd.

It can greatly increase the selectivity of treatment and reduce side effects by specifically turning on sonosensitizer activity at the tumor site. However, most sonosensitizers are almost always on. Very recently, Tan et al. developed a TME‐responsive nanosonosensitizer (TiO_2_@CaP) to enhance SDT efficiency for powerful antitumor immunity.^[^ ^100a^ ^]^ TiO_2_@CaP was prepared by coating a layer of dense calcium phosphate (CaP) on the sonosensitizer TiO_2_. TiO_2_@CaP did not initiate SDT under physiological conditions due to the dense coating of CaP. The CaP shell on TiO_2_@CaP was etched to expose the inner core TiO_2_ to specifically turn on SDT at the slightly acidic tumor site. Moreover, Ca^2+^ release from CaP‐mediated mitochondrial dysfunction by inducing Ca^2+^ overloading. Upon US activation, this cascade process triggered potent ICD to enhance tumor‐specific CTLs activation, and promoted the infiltration CTLs into immunogenic cold tumors (**Figure** **7**A). Importantly, TiO_2_@CaP‐mediated SDT in combination with anti‐PD‐1 elicited strong systemic antitumor immune response, which not only suppressed the primary tumor growth, but also regressed the distant tumors (Figure 7B,C).

In addition, multiple‐responsive strategies based on TME can further improve efficiency and reduce related adverse effects. Matrix metalloproteinase‐2 (MMP‐2) was upregulated in numerous cancers, which can mediate cancer invasion and metastasis by degrading extracellular matrix.^[^ ^101^ ^]^ Therefore, Huang et al. designed a pH and MMP‐2 dual‐responsive nanoparticle for tumor‐targeting codelivery of nanosensitizers and immune checkpoint inhibitors.^[^ ^100b^ ^]^ Specifically, they encapsulated sonosensitizer Ce6 into a core–shell lipid micelle via a nanoprecipitation method and conjugated anti‐PD‐L1 antibody to micelle through MMP‐2‐cleavable peptide linker, where an acid‐labile PEG outer layer was finally coated and P‐aPD‐L1/C was fabricated completely. Similarly, P‐aPD‐L1/C can specifically respond to slightly acidic TME, resulting in the shedding of PEG outer layer. In addition, the peptide linker can be cleaved by the overexpressed MMP‐2 in tumor tissue, resulting in the release of anti‐PD‐L1 antibody (Figure 7D). Ce6‐mediated SDT can further enhance antitumor immunity by inducing ICD under US activation. The internalization efficiency of P‐aPD‐L1/C at pH 6.5+10 × 10^−9^ m MMP‐2 (up to 99.8%) was significantly higher than that at pH 7.4 (only 14.6%), indicating the excellent tumor targeting of P‐aPD‐L1/C (Figure 7E). Encouragingly, P‐aPD‐L1/C induced significant cell death at pH 6.5 + 10 × 10^−9^ m MMP‐2 under US irradiation, with the ratio of cell apoptosis as high as 61.5%. P‐aPD‐L1/C also induced the most remarkable CRT exposure under US irradiation, thus triggering a strong ICD effect to activate antitumor immune responses in vivo, which not only significantly inhibited the growth of primary tumors but also awakened antitumor immune memory to inhibit tumor recurrence (Figure 7F,G), with the minimal systematic immune‐related adverse effects.

## 6 SDT‐Based Multimodal Combination Cancer Immunotherapy

In addition to SDT, other treatments can also induce ICD of cancer cells, such as chemotherapy, CD, PDT, and PTT.^[^ ^102^ ^]^ Multimodal combination therapy has been recognized as a paradigm of current cancer treatment.^[^ ^103^ ^]^ Therefore, considerable efforts have been dedicated to integrate SDT with other therapeutic modalities to synergistically improve the cancer immunotherapy efficacy. These emerging multimodal SDT‐based cancer immunotherapy fall into three main categories: first, multifunctional nanomedicines for chemotherapy and SDT‐based immunotherapy. There is no cross‐resistance between SDT and many chemotherapy drugs. As a result, SDT‐based immunotherapy can be greatly enhanced with appropriate chemotherapeutic agents.^[^ ^104^ ^]^ Second, tailored multifunctional nanomedicines are adopted for combination therapy through CDT/PDT with SDT‐based immunotherapy.^[^ ^105^ ^]^ The biggest bottleneck of SDT is the low yield of ROS. CDT/PDT can significantly increase SDT‐induced ICD by inducing high levels of ROS, thereby enhancing the effect of immunotherapy. Third, combination of SDT‐based immunotherapy and PTT.^[^ ^106^ ^]^ PTT can not only increase the ROS yield of SDT by increasing localized temperature, blood flow, and O_2_ supply at tumor site, but also promote ICD by increasing the expression of HSPs to enhance SDT‐based immunotherapy.

### 6.1 Synergistic SDT/Chemotherapy for Cancer Immunotherapy

Chemotherapy, as an important traditional therapy strategy, has been widely adopted in the treatment of cancer. Tirapazamine (TPZ) is a chemotherapy drug in Phase III clinical trials for head and neck cancer and gynecological cancer, can destroy DNA of tumor cells by generating ROS and benzotriazinyl radicals. More interestingly, TPZ has higher antitumor activity thanks to higher yields of free radicals under hypoxic conditions.^[^ ^107^ ^]^ Recently, Zhang et al. co‐loaded TPZ and chlorin e6 (Ce6) into liposomal nanoparticles (Lipo‐Ce6/TPZ@M_H_) to synergistically enhance SDT‐based cancer immunotherapy by chemotherapy.^[^ ^108^ ^]^ SDT had a strong synergistic therapeutic effect with TPZ: SDT consumed O_2_ to form ROS to promote the efficacy of TPZ by aggravating hypoxia at tumor site, and ROS from TPZ greatly increased SDT‐induced ICD. The surface of Lipo‐Ce6/TPZ@M_H_ was further covered with erythrocyte membrane as biomimetic decoy for the specific tumor targeting and immune escape. Under US, Ce6 triggered SDT to generate a large amount of ROS, hypoxia‐excited TPZ further induced tumor cell apoptosis, achieving efficient synergistic therapy. Importantly, synergistic SDT and hypoxia‐activated chemotherapy directly killed cancer cells to further release large amounts of DAMPs to trigger antitumor immunity, resulting in significant primary melanoma elimination and inhibition of lung metastasis in vivo.

Some widely used clinical chemotherapeutics can induce the effect of ICD, such as oxaliplatin (OXP). Very recently, Zheng et al.^[^ ^104^ ^]^ adopted an emulsification‐solvent evaporation method to prepare shell–core nanostructures (OIX_NPs) with perfluoropentane (PFP) as the core and PLGA loaded with OXP and indocyanine green (ICG) as the shell. Among OIX_NPs, ICG were adopted as photosensitizers and sonosensitizers, and generated ROS through NIR light and ultrasonic; PFP alleviated hypoxia of tumor tissue because of its high O_2_‐carrying capacity; OXP enhanced SDT‐based immunotherapy by inducing ICD effect. Therefore, OIX_NPs increased the effective intratumoral infiltration of CD4 and CD8 T cells, which induced tumor cells apoptosis, necrosis, and autophagy, thereby inhibiting tumor growth. As result, OIX_NPs greatly prolonged median survival of mice with ovarian cancer.

### 6.2 Synergistic SDT/CDT(PDT) for Cancer Immunotherapy

**Figure 8:**
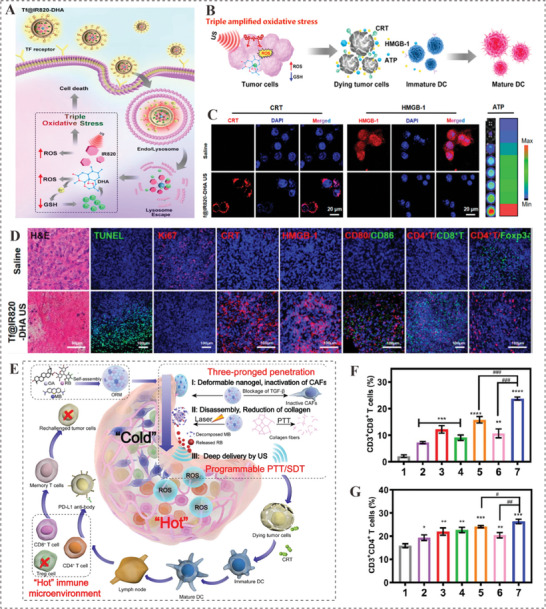
A) Schematic illustration of the mechanism of PIH‐NO on cancer therapy and immune activation. B) Schematic depiction of the mechanism by which ICD‐associated DAMPs in dying tumor cells promote DC maturation. C) CLSM images of Hep1‐6 tumor cells stained by CRT and DAPI or HMGB‐1 and DAPI, and extracellular ATP from Hep1‐6 tumor cells quantified by flow cytometry. D) Representative figures of H&E, TUNEL, Ki67, CRT, HMGB‐1, CD80/CD86, CD4^+^ T/CD8^+^ T, and CD4^+^ T/Foxp3^+^ staining of tumors with different treatments. Adapted with permission.^[^ ^105c^ ^]^ Copyright 2022, American Chemical Society. E) Schematic illustration of the three‐pronged deep penetration of ORM into tumors for synergistic PTT/SDT and anti‐PD‐L1 immunotherapy. F,G) The level of CD8^+^ T cells and CD4^+^ T cells, respectively, in primary tumor tissues obtained from unilateral 4T1 tumor‐bearing mice with different treatment. Adapted with permission.^[^ ^110^ ^]^ Copyright 2022, Elsevier Sci Ltd.

CDT also is an effective strategy to improve SDT‐based immunotherapy efficiency for cancer. For instance, Bai et al. constructed the Tf@IR820‐DHA nanoparticles with excellent tumor targeting for SDT‐based immunotherapy by CDT synergistic therapy.^[^ ^105c^ ^]^ Transferrin (Tf) had a highly specific binding to tumor cells because more than 98% kinds of tumor cells highly expressed Tf receptor. Tf@IR820‐DHA was prepared through two main steps: Tf‐containing 293 T‐cell nanovesicles (Tf NVs) were prepared by genetic engineering method, and then the acid‐responsive ester‐bonded dihydroartemisinin (DHA)‐IR820 (sonosensitizer) conjugates were loaded into Tf NVs. Tf@IR820‐DHA specifically reached the tumor site because of the homologous targeting effect, and DHA‐IR820 were cleaved to release DHA and IR820 in the slightly acidic environment of the tumor. In particular, the abundant Fe^3+^ in Tf were reduced to Fe^2+^ by GSH in cancer cells, which not only impaired the antioxidant capacity of cancer cells, and produced potent CDT due to Fe^2+^ mediated internal peroxide bridges cleavage to promote DHA to form high levels of ROS (**Figure** **8**A). Therefore, Tf@IR820‐DHA significantly enhanced SDT‐induced ICD via CDT synergy under US. The programmable triple oxidative stress of Tf@IR820‐DHA induced the upregulation of CRT and HMGB1 levels in tumor cells and proliferation of CTLs to activate significantly antitumor immune response (Figure 8B–D). Under US irradiation, the Tf@IR820‐DHA fostered the activation and proliferation of CTLs in tumor tissues and further combined with a‐PD‐L1‐mediated ICB to significantly inhibit tumor growth in both primary and distant tumors.

The combination of SDT, CDT, and PDT can further reinforce antitumor therapeutic efficiency due to their different mechanism on generating ROS. Du et al. designed the Pd‐single‐atom coordinated porphyrin‐based polymeric networks (Pd‐Pta/Por) with excellent ROS biocatalytic ability for chem‐/sono‐/photo‐trimodal tumor therapies.^[^ ^109^ ^]^ The Pd‐Pta/Por was synthesized via Pd‐coordination‐driven self‐assembly of PdCl_4_^2−^, tri(pyridine‐4‐yl) amine (Pta), and 5,10,15,20‐tetra(4‐pyridyl)‐21H,23H‐porphine (Por). In the polymeric networks, Pd‐N_2_‐Cl_2_ catalytic center can specifically respond to H_2_O_2_ in TME to generate abundant·OH for CDT, while Por acts as a sono/photosensitizers to produce massive ^1^O_2_ for SDT/PDT under US and laser irradiation. Pta can be used as a modulator to regulate the particle size of Pd‐Pta/Por to endow it with highly uniform size and morphology, which is highly beneficial for subsequent bioapplication. Both in vitro and in vivo results proved that the Pd‐Pta/Por + US + Laser group elicited the most significant cell apoptosis and the highest tumor suppression (up to 90%) than other treatment groups, indicating the potent synergistic CDT, SDT, and PDT performances of Pd‐Pta/Por. Importantly, Pd‐Pta/Por boosted the most significant CRT exposure than other groups to activate antitumor immune responses mediated by ICD.

### 6.3 Synergistic SDT/PTT for Cancer Immunotherapy

PTT is an effective strategy to enhance SDT‐based immunotherapy. For example, Li et al. developed methylene blue (MB, a photothermal agent) based ORM nanogels to improve SDT‐based immunotherapy by PTT.^[^ ^110^ ^]^ The ORM nanogels were prepared by self‐assembly of MB, rose bengal (a sonosensitizer, RB), and oleanolic acid (OA). ORM generated PTT under NIR laser irradiation and triggered the release of RB from the ORM nanogels. Therefore, the ORM nanogels increased the exposure of CRT on the surface of dead cancer cells and induce ICD by synergistical PTT and SDT under the stimulation of US (Figure 8E). Notably, OA acted as an immunomodulator and transforming growth factor‐*β* (TGF‐*β*) antagonist, which inactivated CAFs and remodeled the extracellular matrix. The percentages of CD8^+^ T cells and CD4^+^ T cells were increased to 24.3% and 26.1% in the ORM+L+U mice comparison to that in the control mice (1.71% for CD8^+^ T cells and 16.8% for CD4^+^ T cells), respectively (Figure 8F,G). Importantly, ORM‐mediated PTT/SDT combined with anti‐PD‐L1 checkpoint blockade therapy significantly inhibited the growth of primary and distant tumors in triple‐negative breast cancer (TNBC) T1 tumor model mice. Similarly, Lin et al. loaded hematoporphyrin methyl ether (HMME, as sonosensitizers) and superparamagnetic iron oxide (SPIO, as sonosensitizers) into cancer cell membrane (CCM)‐modified PLGA nanoparticles to prepare biomimetic nanoprobes CHINPs. CHINPs exhibited excellent homologous tumor targeting and PTT enhanced SDT. As a result, CHINPs induced elevation of CD8^+^T cells and decrease of Tregs, as well as the enhanced DC maturation and cytokine secretion, which enhanced anti‐PD‐1 immunotherapy for eliminating primary and metastatic tumors.^[^ ^106a^ ^]^

### 6.4 Synergistic SDT/Gas Therapy for Cancer Immunotherapy

Gas‐mediated cancer therapy has attracted widespread attention due to their high therapeutic efficiency and spatiotemporal specificity.^[^ ^111^ ^]^ Numerous efforts have been devoted to explore new gas therapy and gas‐sensitized synergistic therapy to further enhance anticancer effects, which mainly focus on the precise and controlled release of gases with known therapeutic effect, such as, nitric oxide (NO), carbon monoxide (CO), carbon dioxide (CO_2_), and O_2_. Encouragingly, the effect of SDT can further amplify by these gases therapy and then induce more powerful ICD to activate antitumor responses.

#### 6.4.1 NO/CO‐Enhanced SDT‐Based Cancer Immunotherapy

As the first recognized therapeutic gaseous signaling molecule, NO plays a key role in many physiological and pathological processes.^[^ ^112^ ^]^ However, the precise and controllable release of NO remains a challenge. Stimuli‐responsive NO release in a specific pathological microenvironment provides a strong foundation for the emerging paradigm of precise therapy,^[^ ^113^ ^]^ such as antibacterial treatment,^[^ ^112^, ^113^ ^]^ cancer therapy,^[^ ^114^ ^]^ etc. Light stimulus represents a noninvasive and spatiotemporal strategy for NO release. For example, Wang et al. reported a photoacoustic (PA) cavitation‐enhanced NO release nanoplatform (namely NO‐NCPs) for PA and fluorescence imaging‐guided cancer therapy. The NO‐NCPs can specifically trigger NO release in acidic lysosomes. Importantly, the release of NO can further be accelerated by the PA cavitation generated from pulsed laser irradiation at NIR range. Similarly, US‐triggered NO release is also an effective strategy for cancer therapy. Recently, SDT combined with NO gas therapy has shown excellent synergistic antitumor immune effect because high concentrations of NO can inhibit mitochondrial activity and damage DNA to further promote cancer cell apoptosis.^[^ ^114a^ ^]^ Taking advantage of this, Wang et al. designed a US‐triggered “nanovaccine” like system based on L‐arginine (LA)‐loaded black mesoporous titania (BMT) nanocomposites (BMT@LA NCs).^[^ ^115^ ^]^ The obtained BMT@LA NCs simultaneously activate BMT and LA to produce ^1^O_2_ and release massive NO at the tumor site upon US stimulation to induce severe cancer cell apoptosis. Furthermore, when combined with anti‐PD‐L1 checkpoint blockade therapy, the antitumor effect was significantly improved by the sonodynamic/gas/immune synergistic therapy.

**Figure 9:**
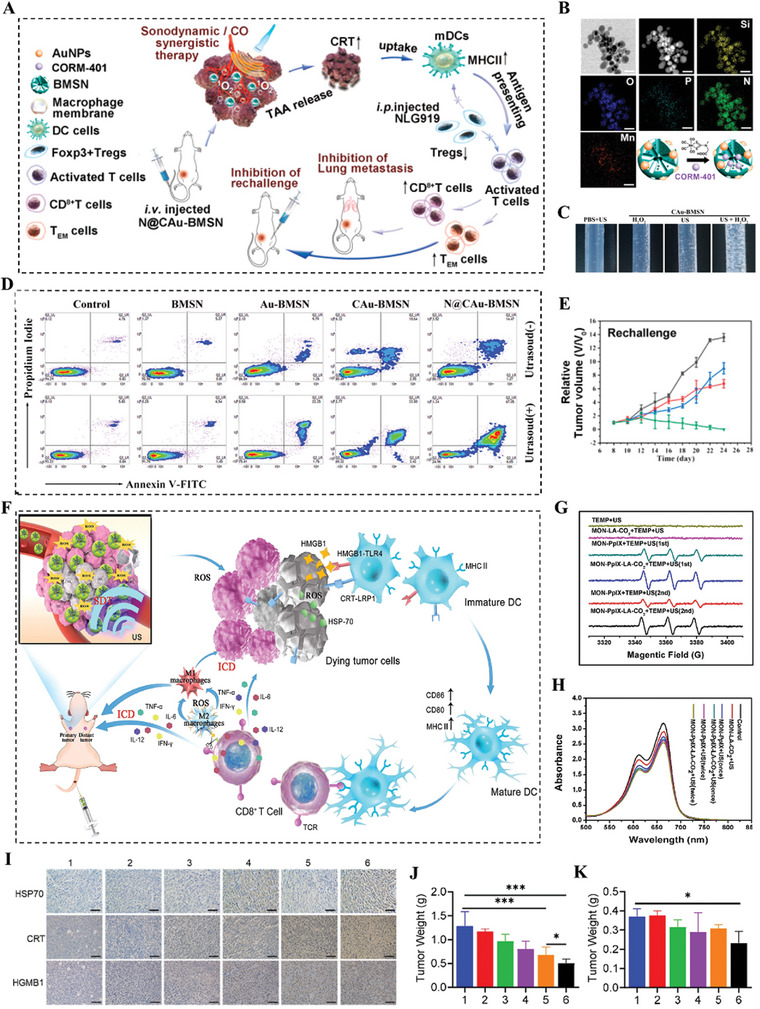
A) Schematic illustration of the prepared biomimetic nanosystem N@CAu‐BMSNs. B) STEM‐HAADF image and the corresponding element mapping images of CAu‐BMSNs. C) Photo images of bubble generation of CO gas. D) Apoptosis of 4T1 cells treated with different doses of BMSNs, Au‐BMSNs, CAu‐BMSNs, and N@CAu‐BMSNs upon US triggering for 60 s (1 MHz, 1 W cm^−2^). E) Rechallenge tumor volume change of mice after receiving indicated treatments with US triggering (*n* = 4). Adapted with permission.^[^ ^118^ ^]^ Copyright 2021, American Chemical Society. F) Schematic illustration of continuous UIC‐enhanced ROS production for promoting ICD‐based immunotherapy process based on this nanoplatform. G) ESR spectra of different groups including TEMP + US, MON‐LA‐CO_2_+TEMP + US, MON‐PpIX + TEMP + US (first), MON‐PpIX‐LA‐CO_2_+TEMP + US (first), MON‐PpIX + TEMP + US (second), and MON‐PpIX‐LA‐CO_2_+TEMP + US (second). H) UV–vis spectra of MB after treatments with different groups for evaluating ·OH production. I) Immunohistochemical examination of HSP70, CRT, and HMGB1 after different treatments in tumor model. J,K) Tumor weight variation of primary tumors (left) and distant tumors (right) on mice after different treatments. Adapted with permission.^[^ ^120^ ^]^ Copyright 2021, Elsevier Sci Ltd.

As we all known, high concentrations of CO can also efficiently induced cancer cell apoptosis by blocking mitochondrial respiration and inhibiting glycolysis.^[^ ^116^ ^]^ However, CO was also highly toxic to normal tissues, and must be specifically released in cancer lesions to achieve therapeutic effects and avoid toxicity.^[^ ^117^ ^]^ Recently, Zhang et al. developed a nano‐sonosensitizer (Au‐BMSNs) loaded with CO precursor (CORM‐401) that specifically released CO at the tumor site to enhance the efficacy of SDT‐induced ICD (**Figure** **9**A).^[^ ^118^ ^]^ Au‐BMSNs were prepared by in situ growth of gold nanoparticles on mesoporous silica loaded with black phosphorus nanoparticles. Au‐BMSNs were efficiently loaded with CORM‐401 due to the high void volume (0.658 cm^3^ g^−1^) and subsequently covered with a layer of macrophage cell membrane (N@CAu‐BMSN) to increase tumor targeting and bypass reticuloendothelial system (RES) evasion abilities (Figure 9B). The high concentration of H_2_O_2_ can accelerate the oxidation of CORM‐401 to generate CO at the tumor site under US irradiation (Figure 9C), which reduced the membrane potential of mitochondria and induced severe cancer cell apoptosis (as high as 75.32%) for improving SDT‐induced ICD effect (Figure 9D). Importantly, by combining with indoleamin 2,3‐dioxygenase (IDO) signal blocking, N@CAu‐BMSN not only effectively induced antitumor immunity by converting memory T cells into effector T cells and reducing Treg cells, but established long‐term immune memory to prevent pulmonary metastasis of tumors (Figure 9F).

#### 6.4.2 CO_2_‐Enhanced SDT‐Based Cancer Immunotherapy

The yield of ROS was mainly determined by the US‐triggered inertial cavitation (UIC) in SDT.^[^ ^119^ ^]^ However, the inertial cavitation quickly disappeared with the cessation of sonication resulting in low ROS yield of nanosensitizers. Recently, Yin et al. reported a MON‐PPIX‐LA‐CO_2_ nanoplatform by co‐encapsulating the sonosensitizer protoporphyrin (PpIX) and l‐arginine (LA) in mesoporous silica nanoparticles for highly efficient SDT‐induced immunotherapy (Figure 9F).^[^ ^120^ ^]^ CO_2_ was efficiently loaded by MON‐PPIX‐LA because of the high affinity guanidine group of LA for CO_2_ under physiological conditions.^[^ ^121^ ^]^ MON‐PPIX‐LA‐CO_2_ continuously formed UIC by releasing CO_2_ bubbles to greatly enhance SDT under a single irradiation of US (Figure 9G), and induced a high yield of ^1^O_2_ and ·OH to induce cancer cell apoptosis (Figure 9H). Importantly, the highly accumulated ROS in tumor cells led a powerful ICD to activate the systemic antitumor immune response. After a single intravenous injection, MON‐PPIX‐LA‐CO_2_ effectively accumulated in tumor tissues thanks to ERP of tumor. In unilateral 4T1 tumor‐bearing model, MON‐PpIX‐LA‐CO_2_+US (twice) group achieved higher level of TAAs exposure and DAMPs (CRT, HSP70 and HMGB1) than control group (Figure 9I). Importantly, the robust antitumor immune effect of MON‐PpIX‐LA‐CO_2_ significantly inhibited primary tumor growth and suppressed distal tumor growth (Figure 9J,K).

#### 6.4.3 O_2_‐Enhanced SDT‐Based Cancer Immunotherapy

Hypoxia is a typical characteristic of TME and can significantly hinder the therapeutic efficiency of SDT. Recently, some well‐designed nanosensitizers have shown great potential in improving the effect of SDT‐induced ICD by relieving the hypoxic microenvironment.^[^ ^122^ ^]^ O_2_ concentration can be efficiently increased by some O_2_ carriers at low O_2_ partial pressures of tumors, such as perfluorocarbons or hemoglobin. For example, Ji et al. developed a human serum albumin‐NO carrier (HSA‐NO) loaded with perfluorodecalin (PDC) and IR780 (PIH‐NO) for enhancing the effect of SDT‐induced ICD by alleviating hypoxia.^[^ ^122a^ ^]^ PIH‐NO was prepared by a two‐step method: HSA‐NO was loaded with a hydrophobic sonosensitizer‐IR780, and then formed nanomicelles. Under US, PIH‐NO continuously provided O_2_ to IR780 to generate ^1^O_2_, which greatly improved the SDT effect due to the high ^1^O_2_ loading capacity of PDC. Moreover, PIH‐NO produced high concentrations of NO under US, and then NO generated strong oxidative peroxynitrite anion together with ^1^O_2_ to further enhance the SDT‐induced ICD effect. More importantly, PIH‐NO reversed the immunosuppressive TME and enhanced tumor immunotherapy by inducing the transformation of M2‐type macrophages to M1‐type, depleting MDSCs, promoting DCs maturation, and increasing T cells infiltration. After intravenous administration, the PIH‐NO exhibited enhanced accumulated in tumors in 4T1 mice due to the increased blood perfusion volume of NO produced by PIH‐NO. PIH‐NO+US treatment significantly enhanced CRT exposure, HMGB1 and ATP release, thereby amplifying ICD. The tumor inhibition rate of PIH‐NO+US treatment was about 92%, which was significantly higher than that of IH+US treatment (27%) and PIH+US treatment (63%) tumor suppression rates. Similarly, Yang et al.^[^ ^122b^ ^]^ developed a multifunctional sonosensitizer (PFCE@THPP_pf_‐COPs) by conjugating sonosensitizer THPP, perfluorosebacic acid (PFSEA), and perfluoro‐15‐crown‐5‐ether (PFCE, a perfluorocarbon molecule). The PFCE@THPP_pf_‐COPs efficiently alleviated the hypoxic of tumors under the low‐frequency US exposure due to the high O_2_ loading capacity of PFCE. The positive hypoxia area percent of tumor slices from these mice with PFCE@THPP_pf_‐COPs treatment was only 6.2%, which was remarkably lower than 20.4% and 19.9% for these PFCE@THPP_pf_ treated and control mice. Furthermore, the combination of PFCE@THPP_pf_‐COPs mediated SDT with anti‐CD47 immunotherapy significantly suppressed tumor growth and triggered potent immunological memory by promoting the tumor infiltration of M1 macrophages and adaptive cytotoxic CD3^+^CD8^+^ T cells, as well as restricted the intertumoral frequencies of Tregs.

It is also an effective strategy for alleviating hypoxia by converting the high concentration of H_2_O_2_ into O_2_ in the TME. For instance, Jiang et al. proposed a “H_2_O_2_‐economizer” strategy for on‐demand H_2_O_2_ decomposition‐assisted O_2_ generation.^[^ ^122c^ ^]^ Ce6 was loaded in catalase‐like Fe‐PDAP (Fe‐doped polydiaminopyridine), and subsequently coated with cancer cell membrane (as a tumor targeting group) to obtain membrane‐coated Fe‐PDAP/Ce6 (MFC). MFC were dissociated to generate robust O_2_ supply by exposing Fe‐PDAP under US irradiation. After intravenous injection, MFC effectively accumulated in tumor tissues due to the specific homologous targeting ability of cancer cell membranes. Under US irradiation, MFC‐mediated SDT promoted DCs maturation and increased the infiltration of CD8^+^ T cells, while decreased the level of Tregs in distant tumors. Importantly, SDT in combination with anti‐PD‐1 antibody had demonstrated superb antitumor performance in both primary tumors and distant tumors.

**Table 3:** Recent advances in SDT‐based cancer immunotherapy enhanced by multiple strategies

Categories	Nanomaterials	Mechanisms	Advantages	Ref.
Enhanced SDT‐induced ICD	Zn‐TCPP/CpG	Synergistic effect of adjuvant cpG and Zn‐TCPP‐mediated SDT to enhance ICD	Enhanced electron conduction; inherent biodegradability	[91a]
	cMn‐MOF@CM	Synergistic effect of adjuvant cpG, Mn‐MOF‐mediated SDT, and anti‐PD‐L1	Prolonged blood circulation; enhanced tumor targeting	[90]
	HMME/R837@Lip	Synergistic effect of HMME‐mediated SDT, R837 and anti‐PD‐L1	High tumor accumulation and prolonged tumor retention; excellent biosafety	[91b]
	Zr‐TCPP(TPP)/R837@M	Synergistic effect of Zr‐TCPP(TPP)‐mediated SDT, adjuvant R837 and anti‐CTLA‐4	Mitochondria‐targeted; high rate of sonosensitizer loading	[91c]
	MnO_2_‐Poly(I:C) @COF NPs	Synergistic effect of COF‐mediated SDT, adjuvant Poly(I:C) and GSH‐responsive Mn^2+^ release; hypoxia relief	Enhanced SDT toxicity and ICD effect; low toxicity and good biocompatibility	[123]
	PEG‐IR‐Mn^2+^‐sabutoclax	Synergistic effect of PEG‐*b*‐IR‐mediated SDT, Sabutoclax enhanced GSH consumption, Mn^2+^ activated cGAS‐STING pathway and anti‐PD‐L1	Significant contribution of the DCs maturation	[92]
	iCRET NPs	Enhanced ROS quantum yield by CRET; CO_2_ bubbles could elicit ICD by rupturing the plasma membrane of cancer cells via acoustic cavitation	TME response; high ROS quantum yield	[98]
TME‐responded SDT‐based immunotherapy	TiO_2_@CaP	TiO_2_‐mediated SDT; pH‐responsive Ca^2+^ release; combined with anti‐PD‐1	“Smart” TME‐activatable nanosensitizers	[100a]
	PEG‐CDM‐aPD‐L1/Ce6	Ce6‐mediated SDT; combined with anti‐PD‐1	pH and MMP‐2 dual‐sensitive nanodrug release; tumor‐targeting	[100b]
SDT‐based multimodal combination immunotherapy	Lipo‐Ce6/TPZ@M_H_	Synergetic SDT/chemotherapy for antitumor immunotherapy	High‐effective synergistic therapy	[108]
	THPP‐Oxa (IV)‐PEG	Synergetic SDT/chemotherapy	GSH‐responsive chemotherapeutic drug release	[105a]
	OIX_NPs	Synergetic SDT/PDT/chemotherapy for antitumor immunotherapy	Enhanced tumor immunogenicity	[104]
	OI_NPs	Synergetic SDT/PDT/chemotherapy for antitumor immunotherapy	Enhancing immunological potency; dual‐mode imaging guided therapy	[124]
	Tf@IR820‐DHA	Synergetic SDT/CDT/anti‐PD‐L1 for antitumor immunotherapy	Enhanced ROS yield; tumor‐specific targeting; good biocompatibility	[105c]
	PEGylated CoFe_2_O_4_ nanoflowers (CFP)	Synergetic SDT/CDT/anti‐PD‐L1 for antitumor immunotherapy	Multiple enzymatic activities; efficient electron–hole pair separation	[105d]
	TIR@siRNA	Synergistic SDT/gene/anti‐PD‐L1 for antitumor immunotherapy	Gene‐enhanced nuclear targeting	[125]
	Peptide amphiphile‐Rose bengal (RB) nanocapsules (PARN)	Synergistic SDT/PDT for antitumor immunotherapy	Tumor targeting; amphiphilicity	[105b]
	Pd‐Pta/Pro	Synergistic CDT/SDT/PDT for antitumor immunotherapy	High ROS yield; good biocompatibility and biosafety	[109]
	pCN/Ce6	Synergistic SDT/PDT/PTT for antitumor immunotherapy	Low biotoxicity; rapid clearance	[126]
	ORM	Synergistic SDT/PTT/anti‐PD‐L1 for antitumor immunotherapy	Safe; green	[110]
	CHINPs	Synergistic SDT/PTT/anti‐PD‐1 for antitumor immunotherapy	Multimodal imaging‐guided triple therapeutic nanoplatform	[106a]
	PdPt@GOx/IR780	Synergistic SDT/PTT/starvation therapy for antitumor immunotherapy	High‐efficient photothermal properties; significant synergistic effect	[122d]
	N@CAu‐BMSN	Synergistic SDT/gas therapy/IDO signal blocking for antitumor immunotherapy	Effective tumor homing and RES evasion; significant ICD induced by ^1^O_2_ and CO	[118]
	BMT@LA NCs	Synergistic SDT/gas therapy/anti‐PD‐L1 for antitumor immunotherapy	Significant ICD induced by ^1^O_2_ and NO gas	[115]
	MON‐PPIX‐LA‐CO_2_	Continuous CO_2_ bubbling‐enabled UIC to enhance SDT‐induced ICD	High ROS quantum yield; enhanced SDT triggered powerful ICD	[120]
	IRO@FA NPs	IR780‐mediated SDT; PHF‐enhanced O_2_ supply	Tumor targeting	[127]
	PIH‐NO	IR780‐mediated SDT; PFC‐enhanced O_2_ supply; NO‐enhanced oxidative stress	Explosive ROS release; mitochondrial targeting	[122a]
	PFCE@THPP_pf_‐COPs	High efficacies of tumor hypoxia relief; combined with anti‐CD47 immunotherapy	Excellent sound sensitization and TME‐responsive ability	[122b]
	Membrane‐coated Fe‐PDAP/Ce6 (MFC)	On‐demand O_2_ supply; combined with aPD‐1	On‐demand O_2_ supply of US‐activated and tumor targeting	[122c]

## 7 Summary and Outlook

In this review, we systematically summarized the latest research progress of SDT‐based cancer immunotherapy in recent years. These findings suggested that SDT‐based immunotherapy has broad application prospects in the field of tumor therapy, because SDT can not only kill cancer cells through US‐triggered ROS, but also activate host antitumor immune responses by inducing ICD to achieve simultaneous suppression or even elimination of orthotopic and metastases tumors. Notably, SDT‐based multifunctional nanoplatforms allow multiple pathways to amplify antitumor immune effects, such as enhancing SDT‐induced ICD, modulating immunosuppressive TME, relieving tumor hypoxia, and comminating SDT with chemotherapy, CDT/PDT, and PTT. These diverse strategies further expand the application of SDT‐based immunotherapy and facilitate its clinical translation. Although SDT‐based immunotherapy has achieved many encouraging results in the preclinical research stage, there are still some critical concerns and challenges remain to be addressed for its future clinical translation.

**Figure 10:**
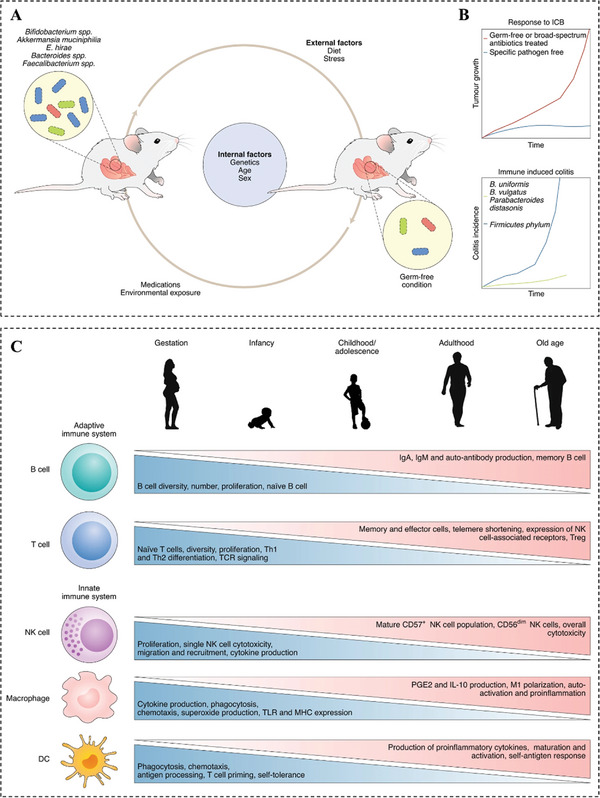
A) The mouse gut commensal microbiota is influenced by multiple internal and external factors. Internal factors include animal genetic makeup, age, and sex. External factors include diet, stress, exercise, environmental exposures, and medication (antibiotics) use. B) Mice housed in different pathogen‐free conditions may show profoundly different responses to ICB. Similarly, the abundance of specific microbial flora is associated with a protective effect on immune‐induced adverse events. C) Human aging is associated with many changes in the immune system. In the bone marrow, hematopoietic cells begin to shift from lymphoid toward myeloid lineages. Naïve T cell numbers continue to drop while more mature cells begin to rise. More inflammatory cytokines are produced, increasing the risk of auto‐antigen responses. Antigen processing and presentation are diminished in dendritic cells, resulting in decreased T cell proliferation and effort molecule expression. Adapted with permission.^[^ ^129^ ^]^ Copyright 2021, Nature Portfolio.

First, the limits of tumor models raise the challenges to the credibility of efficacy and safety outcomes of nanomedicines. An ideal tumor model should recapitulate the features of specific organs harboring tumors, including physiopathological features, TME, microbial flora, aging process, and so on. However, tumor model established in humanized mouse is widely used to evaluate the efficacy of SDT‐based immunotherapy with nanomedicines, which is a kind of ectopic tumor model that fails to replicate various features like physiological barriers. Therefore, there is a huge difference between this model and the pathology of cancer patients, which is also an important contributor for the poor clinical performance of nanomedicines with significant preclinical efficacy. Many current approaches have been proposed to address this bottleneck. For example, genetically engineered mouse is developed to promote tumorigenesis in specific organs, which forms similar physiologically relevant characteristics in vivo. But species divergence between humans and animals still cannot be ignored.^[^ ^128^ ^]^ Emerging human tumor organoid can effectively reproduce microenvironment and other features, which is expected to be a promising surrogate of animal models for nanomedicines screening but still has a long way to go. In addition, an important point is that the microbial flora profoundly influences the therapeutic benefits of immunotherapy (**Figure** **10**A,B).^[^ ^129^ ^]^ For example, the gut microbial diversity of patients that are responsive to immunotherapy is reported to be higher than patients that do not respond to immunotherapy.^[^ ^130^ ^]^ Therefore, the microbiota status can be better indicated in animal tumor model by exposing laboratory animals to bacteria existed in environments like contact with pets. Of note, current mice models are generally established in their youth (6–8 weeks, equivalent to 11 years of age in humans), which is significantly different from the most common age range of cancer patients (generally occurring over 50 years old). Aging can significantly lead to the degeneration of the immune system resulting in low anti‐infection and antitumor capabilities (Figure 10C).^[^ ^131^ ^]^ Therefore, aged mice are more likely to favor the screening and researches of SDT based nanomedicines for immunotherapy.

**Figure 11:**
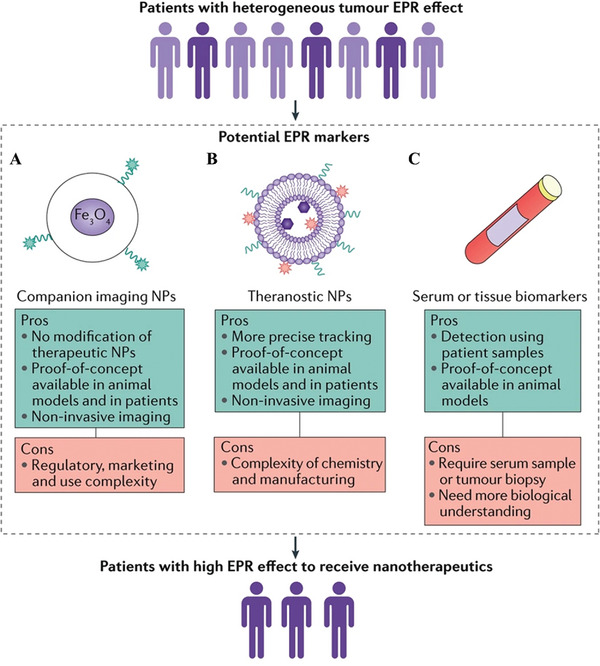
A) Companion imaging agents (e.g., ferumoxytol nanoparticle [NP]) have been applied to predict the accumulation of poly(d, l‐lactic‐*co*‐glycolic acid)‐*b*‐poly(ethylene glycol) (PLGA‐PEG) NP‐encapsulated docetaxel and its anticancer activity in solid tumors, and ferumoxytol is currently in clinical trials to determine its feasibility as a predictive marker for the liposomal irinotecan MM‐398. B) Theranostic NPs have been used to monitor their biodistribution and tumor accumulation using various imaging techniques both preclinically and clinically. C) Serum and tissue biomarkers may also serve as surrogate markers for the enhanced permeability and retention (EPR) effect, as suggested by one recent example showing strong correlation of liposome accumulation in tumors with the relative ratio of matrix metalloproteinase 9 (MMP9) to tissue inhibitor of metalloproteinase 1 (TIMP1) in the circulation. Adapted with permission.^[^ ^132^ ^]^ Copyright 2021, Nature Portfolio.

Second, the heterogeneity of EPR effect in cancer patients impair the advantages of many nanomedicines.^[^ ^132^ ^]^ Nanomedicines have been widely used to cancer therapy based on the EPR effect of solid tumors. However, the heterogeneity of EPR effect is also responsible for the poor efficacy of valuable nanomedicines in cancer patients. Targeting cancer cells and inducing related ICD are essential for SDT to eliminate tumors. Therefore, SDT‐based immunotherapy with nanomedicines is also hampered by the heterogeneity of EPR effect. Patients with obvious EPR can be effectively screened through the stratification of cancer patients, so that SDT‐based immunotherapy can be carried out precisely, which can greatly improve the treatment effect and avoid unnecessary risks and drug resistance. For example, EPR effect in solid tumors can be assessed by magnetic resonance imaging (MRI) nanoprobes (**Figure** **11**A),^[^ ^133^ ^]^ and radioisotope diagnostic nanoprobes (Figure 11B).^[^ ^134^ ^]^ In addition, some blood markers are also highly related to the EPR effect of solid tumors, such as MMP9, tissue inhibitor of metalloproteinase 1, fibroblast growth factor 2, and so on.^[^ ^135^ ^]^ The EPR effect in patients can also be assessed and stratified by detecting these biomarkers (Figure 11C).

Third, great attention should be paid to further enhancing the therapeutic efficiency of SDT‐based immunotherapy. In terms of sonosensitizers, limited ROS yield, and low tumor‐targeting greatly hinder the effect of SDT and subsequent antitumor immune responses induced by ICD. Amplifying acoustic cavitation effect (e.g., CO_2_ bubbling‐enabled UIC), engaging extra energy supply (e.g., CRET), depleting GSH, and combined with other ROS‐mediated therapies (such as chemotherapy, PDT, CDT) are effective strategies to augment ROS production and elicit stronger ICD effect. To improve tumor targeting, TME‐responsive components, antibodies/peptides that bind to specific receptors overexpressed on cancer cells, and biomimetic cell membranes are highly desirable to be introduced to SDT‐based nanomedicines. Nevertheless, SDT‐based immunotherapy efficacy still suffers from the complex TME, such as severe hypoxia and immunosuppressive microenvironment. Deficient O_2_ supply and continuous O_2_ consumption not only limit the efficiency of SDT, but also inhibit the infiltration of CTLs to tumor tissues, greatly suppressing antitumor immune responses triggered by ICD. To overcome this dilemma, exogenous O_2_ supply or endogenous O_2_ generation, combination with hypoxia‐activated chemotherapy or PTT, and directly blocking HIF‐1 pathway are effective enhanced strategies to alleviate tumor hypoxia. In a word, exploring and developing more targeted and efficient SDT‐based immunotherapy strategies is a basic prerequisite for its clinical translation.

Fourth, the optimization of US parameters and scale manufacturing of nanomedicines need to be considered. The physical properties of US, like intensity and pulse frequency, are responsible for the efficacy of SDT. The formation of the standing wave by the interference of ultrasonic waves can also greatly improve the effect of SDT in the tumor site. The optimization of US equipment and SDT parameters can greatly improve the effect of SDT‐based immunotherapy. In addition, US‐based nanomedicines become more and more complex with multiple components to enhance the efficacy of SDT and overcome the limitations of TME. In general, large‐scale and reproducible synthesis is more difficult when NP formulations involve multiple steps or complex techniques. The standardization of scalable manufacturing may be effective to solve this problem.

Finally, the long‐term toxicity and biosafety of SDT‐based nanomedicines in vivo always has been a close concern in this field. At present, the metabolism and long‐term toxicity studies of these nano‐immunoagents are mostly confined to one month or even less, further exploration of their long‐term toxicity and metabolic pathways in vivo is needed. Therefore, exploring nanosonosensitizers with good biocompatibility, biodegradability, and removability through advanced nanoengineering techniques is very beneficial for the clinical translation of SDT‐based immunotherapy.

In summary, SDT‐based immunotherapy is a new cancer treatment strategy with great research value and clinical application prospects, which is expected to become a potential alternative to traditional cancer therapies such as surgery, radiotherapy, and chemotherapy. With the continuous and rapid development of nanomedicines, we believe that SDT‐based cancer immunotherapy will achieve clinical translation sooner, bring Gospel to most cancer patients.

## Conflict of Interest

The authors declare no conflict of interest.

## Author Contributions

Y.Y. and J.H. contributed equally to this work. All authors listed have made a substantial, direct, and intellectual contribution to the work.
